# Increased spontaneous activity of the superior frontal gyrus with reduced functional connectivity to visual attention areas and cerebellum in male smokers

**DOI:** 10.3389/fnhum.2023.1153976

**Published:** 2023-03-17

**Authors:** Xiaoyu Niu, Xinyu Gao, Qingqing Lv, Mengzhe Zhang, Jinghan Dang, Jieping Sun, Weijian Wang, Yarui Wei, Jingliang Cheng, Shaoqiang Han, Yong Zhang

**Affiliations:** ^1^Department of Magnetic Resonance Imaging, The First Affiliated Hospital of Zhengzhou University, Zhengzhou, Henan, China; ^2^Key Laboratory of Magnetic Resonance and Brain Function of Henan Province, Zhengzhou, China; ^3^Engineering Technology Research Center for Detection and Application of Brain Function of Henan Province, Zhengzhou, China; ^4^Key Laboratory for Functional Magnetic Resonance Imaging and Molecular Imaging of Henan Province, Zhengzhou, China; ^5^Engineering Research Center of Medical Imaging Intelligent Diagnosis and Treatment of Henan Province, Zhengzhou, China; ^6^Zhengzhou Key Laboratory of Brain Function and Cognitive Magnetic Resonance Imaging, Zhengzhou, China; ^7^Henan Key Laboratory of Imaging Intelligence Research, Zhengzhou, China; ^8^Henan Engineering Research Center of Brain Function Development and Application, Zhengzhou, China; ^9^Department of Radiology, The Third Affiliated Hospital of Zhengzhou University, Zhengzhou, China

**Keywords:** amplitude of low frequency fluctuation (ALFF), functional connectivity (FC), superior frontal gyrus, visual attention, cigarette smoking

## Abstract

**Background:**

Chronic smokers have abnormal spontaneous regional activity and disrupted functional connectivity as revealed by previous neuroimaging studies. Combining different dimensions of resting-state functional indicators may help us learn more about the neuropathological mechanisms of smoking.

**Methods:**

The amplitude of low frequency fluctuations (ALFF) of 86 male smokers and 56 male non-smokers were first calculated. Brain regions that displayed significant differences in ALFF between two groups were selected as seeds for further functional connectivity analysis. Besides, we examined correlations between brain areas with abnormal activity and smoking measurements.

**Results:**

Increased ALFF in left superior frontal gyrus (SFG), left medial superior frontal gyrus (mSFG) and middle frontal gyrus (MFG) as well as decreased ALFF in right calcarine sulcus were observed in smokers compared with non-smokers. In the seed-based functional connectivity analysis, smokers showed attenuated functional connectivity with left SFG in left precuneus, left fusiform gyrus, left lingual gyrus, left cerebellum 4 5 and cerebellum 6 as well as lower functional connectivity with left mSGF in left fusiform gyrus, left lingual gyrus, left parahippocampal gyrus (PHG), left calcarine sulcus, left cerebellum 4 5, cerebellum 6 and cerebellum 8 (GRF corrected, Pvoxel < 0.005, Pcluster<0.05). Furthermore, attenuated functional connectivity with left mSGF in left lingual gyrus and PHG displayed a negative correlation with FTND scores (*r* = −0.308, *p* = 0.004; *r* = −0.326, *p* = 0.002 Bonferroni corrected).

**Conclusion:**

Our findings of increased ALFF in SFG with reduced functional connectivity to visual attention areas and cerebellum subregions may shed new light on the pathophysiology of smoking.

## 1. Introduction

Cigarette smoking is among the leading causes of preventable illness and death in the earth, characterized by craving, relapsing and withdrawal symptoms (Asma et al., 2014). It is closely related to a variety of tumors, respiratory and digestive system diseases, and adversely affects human health, economics, and life (Goldfarbmuren et al., 2020; Yang et al., 2022). According to statistics, smoking contributes to roughly one in five deaths (Jamal et al., 2018). While many smokers know the adverse consequences of long-term smoking, relapse rates are higher than 85 percent among those who attempt to stop smoking by themselves (Weiland et al., 2015). Given the serious harms of smoking, understanding the neural mechanisms underlying it may contribute to guide brain-based smoking cessation treatments.

The resting state functional magnetic resonance imaging (rs-fMRI) technique, which measures brain activity under resting state using low-frequency fluctuations of the blood oxygen level dependent (BOLD) signal (Biswal et al., 1995), has been extensively employed in the study of the abnormalities of neural activity in a wide range of neuropsychiatric diseases (Zhang et al., 2019; Zhou et al., 2019; Dong et al., 2021; He et al., 2021; Han et al., 2022). Regional spontaneous neural features have shown to be significant for comprehending the neuropathologic and neurophysiological condition of disease (Raichle, 2006). Spontaneous low-frequency fluctuation of BOLD signal has been certified to be closely related to the spontaneous activity of neurons (Lu et al., 2007; Mantini et al., 2007). Amplitude of low frequency fluctuation (ALFF) served as a common approach characterizes the spontaneous regional neural activity by measuring the intensity of spontaneous fluctuation areas (Zang et al., 2007; Wang et al., 2021). Many researchers measured spontaneous brain activity in substance users by using ALFF and found that it is influenced by substance abuse (e.g., cigarettes, heroin, alcohol) (Brody, 2006; Jiang et al., 2011). Studies on humans and animals have confirmed that nicotine disturbs brain regional spontaneous activity, which is linked to the dopaminergic system and nicotinic acetylcholine receptors (Hyman et al., 2006). It has widely reported that brain activity globally reduced in acute administration of nicotine (Brody, 2006). Chronic smokers were also reported to have altered spontaneous brain activity during resting state in some studies (Yu et al., 2013; Chu et al., 2014; Wu et al., 2015; Wen et al., 2021). Wen et al. (2021) has found that smokers showed significantly increased static ALFF in the bilateral middle frontal gyrus and left inferior frontal gyrus. Increased dynamic ALFF in the left inferior frontal gyrus, left superior/medial frontal gyrus and bilateral middle frontal gyrus have also been reported in smokers than non-smokers. In addition, temporal variability of dynamic ALFF in the left superior/medial frontal gyrus and right middle frontal gyrus showed positive correlations with smoking measurements such as pack-years and Fagerström Test for Nicotine Dependence (FTND) scores. Increased spontaneous regional activity in the prefrontal cortex involved in cognitive control and reward processing were previous most commonly findings (Domino et al., 2000; Wen et al., 2021), which is an important target neural region for smoking addiction.

Amplitude of low frequency fluctuationsALFF allows us to know the amplitude of regional neuronal activity, potentially identifying abnormal local functional brain activity. In addictive diseases, more than just the spontaneous regional activity within specific brain regions drives the disease, but also coordination between them (Wang et al., 2018). Whether the functional connections between brain regions with abnormal spontaneous regional activity and other regions are altered in chronic smokers remains unexplored. Using resting-state functional connectivity (rs-FC) method, we can measure temporal correlation between BOLD signals in specific brain networks, finding the degree of interregional cooperation between various spatial regions. There was widespread FC abnormality in certain brain regions among smokers in comparison with non-smokers, according to previous studies. Wang has found decreased FC of left thalamo-precuneus in relapsing smokers (Wang et al., 2020). In smokers as opposed to non-smokers, FC has been found to be attenuated in the reward circuit (Shen et al., 2016). Furthermore, the relationship between altered FC of the brain and the severity of nicotine dependence in smokers has been investigated by many neuroimaging studies. The severity of smoking was usually measured using FTND scores (Heatherton et al., 1991). An extensive study has reported a positive correlation between FTND scores and enhanced FC in insular and visual processing cortex in smokers (Claus et al., 2013). Therefore, it is crucial to explore various aspects of resting state functioning to elucidate the neural mechanisms of smoking, since there may be differences in both local neuronal activity and connectivity patterns.

In this research, we first calculated the spontaneous regional brain activity of two groups (smokers and non-smokers) by using the ALFF. Then, brain regions that displayed significant differences in ALFF between smoking group and control group were used as seeds for further seed-based FC analysis. At last, we performed correlation analyses between brain areas with abnormal activity and smoking-related behavior. Based on previous functional research of smoking, we predicted that chronic smokers would show increased spontaneous regional activity in cognitive control network and FC analyses would reveal disrupted functional connectivity between the seeds and some regions of the brain intimately connected to their function.

## 2. Materials and methods

### 2.1. Participants

This study included 142 male participants aged 25–45 (86 smokers and 56 non-smokers) from a local hospital as well as via the Internet. Since the smoking rate is much higher among Chinese men than women, the public health burden falls mainly on this group (He et al., 2020). The current study focused on male smokers. The severity of cigarette smoking was measured by FTND scores, and pack-years was calculated as (years of smoking multiplied by the number of cigarettes smoked per day)/20 (Thomas, 2014). Smokers were included by meeting the following criteria: (1) right-handed; (2) meeting the diagnosis criteria for substance use disorder in the fifth edition of the Diagnostic and Statistical Manual for Mental Disorders (DSM-5); (3) smoking no less than 10 cigarettes per day and continue for at least 2 years (Zhang et al., 2022); (4) smokers did not attempt to stop smoking and had no abstinence history. The inclusion criteria for non-smokers were:(1) smoking less than five cigarettes in their lifetime; (2) age, gender, and education level were matched to the smokers group; (3) none of the included controls had been exposed to long-term second-hand smoke. Exclusion criteria included any physical illness including obstructive lung disease, epilepsy, brain tumor or cerebrovascular disease; addiction to other substance (except nicotine); a history of neuropsychiatric disease; contraindications to MRI. The study has been approved by the Local Medical Ethics Committee of the First Affiliated Hospital of Zhengzhou University. Informed consent was acquired from all study subjects.

### 2.2. MRI data acquisition

Magnetic resonance imaging data were acquired using a MAGNETOM Skyra 3T MR scanner (Siemens Healthcare, Erlangen, Germany) with 64 channel head coils. Smokers were required to smoke a cigarette 30 min before the scan to avoid nicotine withdrawal symptoms. During the scanning, each participant was asked to close their eyes, not to think of anything special, breathe quietly, and avoid falling asleep. At the end of the scan, the subjects were asked if they had fallen asleep, and those who had fallen asleep were eliminated. We used foam pads and earplugs to minimize head movement and canner noise. The following echo planar imaging parameters were used to acquire functional images: repetition time (TR)/echo time = 2,000/30 ms, matrix size = 64 × 64, flip angle = 80°, field of view = 240 × 240 mm, voxel size = 3 mm × 3 mm × 3 mm, slices = 36, slice thickness = 4 mm, and 180 volumes in total.

### 2.3. Data analysis

This functional imaging data set was preprocessed by using Data Processing Assistant for Resting-state fMRI Analysis Toolkit (DPARSF). Main pre-processing steps and parameters are as follows: (1) conversion of data formats; (2) first five time points were removed due to the instabilities of the initial rs-fMRI signal and patients’ inability to adapt to acquisition conditions initially; (3) slice timing; (4) realignment (excluding subjects with a maximum head motion >2.5 mm or head rotation>2.5°). No subjects were excluded from this step; (5) normalization was performed using EPI templates and resampling was done using 3 mm × 3 mm × 3 mm samples; (6) regression of 24 head motion parameters, global signal, cerebrospinal fluid signal as well as white matter signal (Friston et al., 1996); (7) Frame wise displacement (FD) was calculated for each time point (Power et al., 2012), and an average FD greater than 0.5 mm was excluded; (8) a full-width Gaussian kernel with a half-maximum of 6 mm was used for smoothing; (9) for eliminating low frequency drift and high frequency noise effects, linear trends and temporal filtering (bandpass, 0.01–0.08 Hz) are removed.

Based on Fast Fourier transform (FFT), ALFF was calculated for each voxel and its time series was converted to frequency domain without a band-pass filter. First, the square root was computed at each frequency of the power spectrum, and then the average square root of each voxel in the 0.01–0.08 Hz band was acquired. The last step was to divide the ALFF of each voxel by the global mean of ALFF values (mALFF) (Zang et al., 2007). The resulting ALFF values for each voxel were used for further analysis.

The region of interest (ROI) seed-based method was applied to assess differences in rs-FC between smokers and non-smokers. According to the results of ALFF, brain regions with significant differences in ALFF values between the smoking group and the control group were selected as ROI, which was defined as a sphere with a radius of 5 mm and centered on the peak coordinates. The Pearson correlation between the average time series of the seed region and the time series of all voxels within the whole brain was calculated to produce a rs-FC map for each subject. Correlation coefficients were transformed to z-scores using the Fisher Z-transform to fit the data to a normal distribution.

### 2.4. Statistical analyses

We used two-sample *t*-tests based on IBM SPSS Statistics software (version 26.0) to compare demographic and clinical characteristics between smokers and non-smokers. As covariates, age, years of education and mean FD were used in a two-tailed two-sample *t*-test to compare ALFF and ALFF-based rs-FC between two groups (Gaussian random field theory GRF corrected, P_voxel_ < 0.005, P_cluster_ < 0.05) based on the MATLABSPM12 toolkit. In order to examine the association between brain regions with abnormal activity and clinical characteristics of smoking, we performed correlation analyses between the values of two functional indicators and smoking measurements (such as FTND scores and pack-years).

## 3. Results

### 3.1. Participant characteristics

In all, 86 male smokers and 56 male non-smokers were recruited in the current study. No significant differences were found in years of education and age between the two groups (all *P*-values>0.05). See Table 1 for details.

**TABLE 1:** Demographics and clinical characteristics.

	Smokers (86)	Non-smokers (56)	*P*-value
Age, y	36.03 ± 7.870	33.96 ± 7.193	0.115
Education, y	14.26 ± 2.460	14.70 ± 2.508	0.305
FTND	4.91 ± 1.932	−	−
Pack-years	21.64 ± 9.72	−	−

All data are reported with mean ± standard deviation; y, years; FTND, Fagerström Test for Nicotine Dependence; Peak-years (years of smoking * cigarettes smoked per day)/20.

### 3.2. Between-group comparisons of ALFF

**FIGURE 1:**
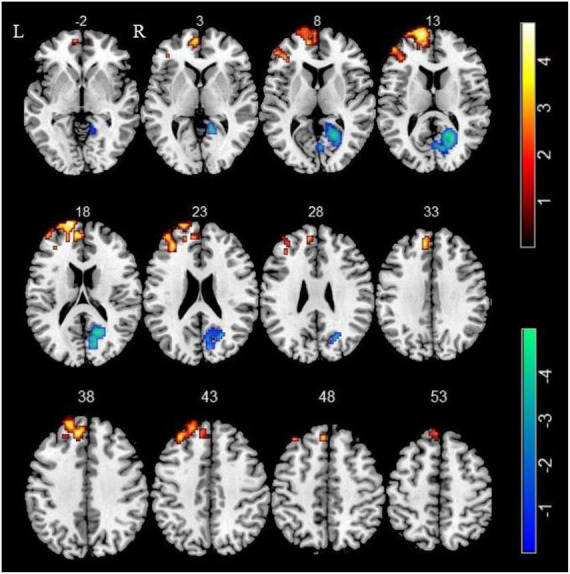
Compared with non-smokers, smokers showed significant differences in ALFF. Regions of increased ALFF were shown in red. Regions of decreased ALFF were shown in blue.

In comparison of the control group, smokers displayed higher ALFF in left superior frontal gyrus (SFG), left medial superior frontal gyrus (mSFG) and left middle frontal gyrus (MFG) as well as displayed lower ALFF in right calcarine sulcus (GRF corrected, P_voxel_ < 0.005, P_cluster_ < 0.05) (Table 2 and Figure 1).

**TABLE 2:** Brain regions with significant differences in ALFF between smokers and non-smokers.

Brain regions	L/R	Peak MNI coordinates	Number of cluster voxels	*t*-values
		(x, y, z)		
**Smokers < non-smokers**				
Calcarine sulcus	R	21, −60, 12	156	-5.00
**Smokers > non-smokers**				
SFG	L	−15, 66, 15	122	4.75
mSFG	L	−9, 42, 37	142	4.12
MFG	L	−33, 42, 24	51	3.60

FG, superior frontal gyrus; mSFG, medial superior frontal gyrus; MFG, middle frontal gyrus; L, left; R, right.

### 3.3. Between-group comparisons of ALFF-based rs-FC

**FIGURE 2:**
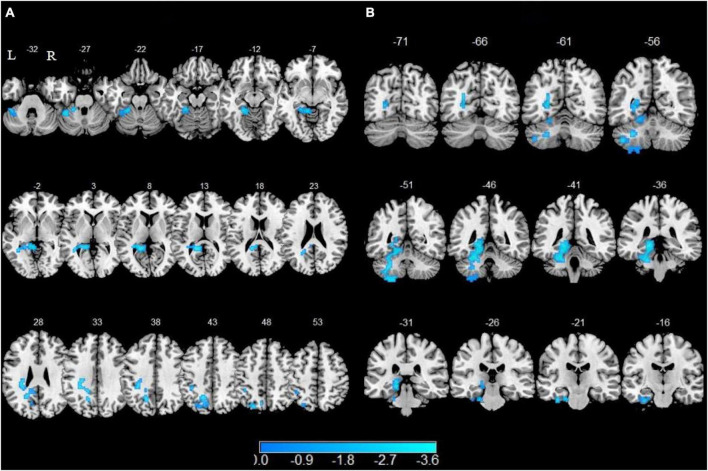
Altered resting-state functional connectivity with the left superior frontal gyrus (SFG) and left medial superior frontal gyrus (mSFG). Brain regions with decreased functional connectivity to the left SFG are shown in panel **(A)**. Brain regions with decreased functional connectivity to the left mSFG are shown in panel **(B)**.

In comparison of the non-smokers, smokers showed attenuated functional connectivity with left SFG in left precuneus, left fusiform, left lingual gyrus, left cerebellum 4 5 and cerebellum 6 as well as lower functional connectivity with left mSGF in left fusiform, left lingual, left parahippocampal gyrus (PHG), left calcarine sulcus, left cerebellum 4 5, left cerebellum 6 and left cerebellum 8 (GRF corrected, P_voxel_ < 0.005, P_cluster_ < 0.05) (Table 3 and Figure 2).

**TABLE 3:** Seed locations and regions showing significant differences in connectivity between smokers and non-smokers.

ROI	Peak MNI coordinates	Number of cluster voxels	*t*-values	Regions
	(x, y, z)			
**R calcarine sulcus**				
**None**				
**L SFG**				
	−18, −48, 9	43	−4.05	L precuneus
	−20, −38, −26	42	−3.09	L cerebellum 4 5
	−37, −46, −26	28	−3.34	L cerebellum 6
	−18, −43, −13	41	−3.13	L fusiform gyrus
	−12, −42, 2	32	−3.69	L lingual gyrus
**L mSFG**				
	−17, −44, −12	77	−3.72	L fusiform gyrus
	−27, −45, −22	77	−3.47	L cerebellum 4 5
	−21, −59, −17	48	−2.68	L cerebellum 6
	−30, −48, −42	43	−3.16	L cerebellum 8
	−16, −39, −10	57	−3.45	L lingual gurus
	−16, −36, −8	37	−3.46	L parahippocampal gyrus
	−24, −60, 11	31	−3.30	L calcarine sulcus
**MFG**				
**None**				

L, left; R, right; SFG, superior frontal gyrus; mSFG, medial superior frontal gyrus; MFG, middle frontal gyrus.

### 3.4. Correlation analysis

**FIGURE 3:**
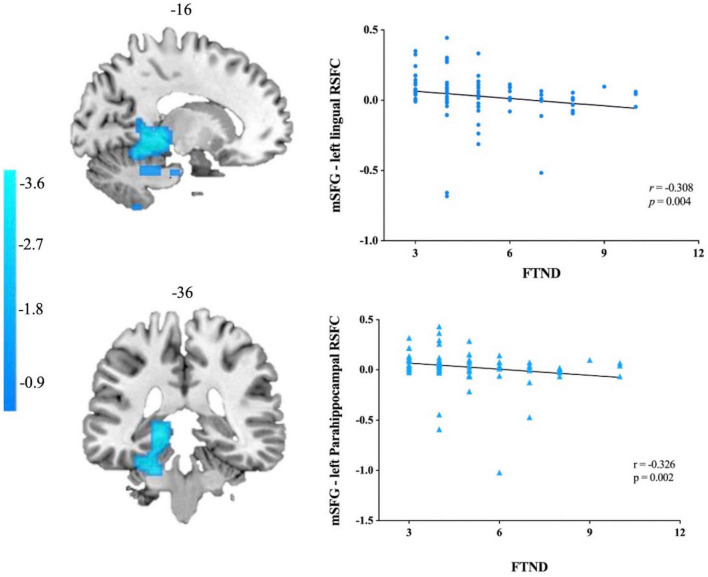
Correlations between mSFG–left lingual gyrus/parahippocampal gyrus rs-FC and the FTND scores in smokers. mSFG, medial superior frontal gyrus; FTND, Fagerström Test for Nicotine Dependence.

The results demonstrated that attenuated functional connectivity with left mSGF in left lingual gyrus and left PHG displayed a negative correlation with FTND scores (*r* = −0.308, *p* = 0.004; *r* = −0.326, *p* = 0.002 Bonferroni corrected). Other brain regions were either not significantly associated with FTND scores or the results did not pass the correction (Figure 3).

## 4. Discussion

Our study combined amplitude of low frequency fluctuation (ALFF) and resting-state functional connectivity (rs-FC) to identify the alterations of intrinsic brain activity and neural connectivity abnormalities in smokers. Two key findings emerged from the study. On the one hand, smokers displayed higher spontaneous regional activity in the prefrontal cortex (PFC) including left superior frontal gyrus (SFG), medial superior frontal gyrus (mSFG) and middle frontal gyrus (MFG) as well as lower spontaneous brain activity in right calcarine sulcus. On the other hand, smokers displayed lower functional connectivity between the PFC with precuneus, parahippocampal gyrus (PHG), cerebellar subregions (such as cerebellum 4 5, cerebellum 6 and cerebellum 8) as well as visual attention areas including the calcarine sulcus, lingual gyrus and fusiform gyrus. Functional connectivity of the mSFG with the PHG and lingual gyrus was negatively related to FTND scores based on correlation analyses. These findings contributed to understand the altered intrinsic brain activity and neural connectivity abnormalities in smokers and highlighted the role of visual attention areas and cerebellum in cigarette smoking.

The ALFF measures the intensity of the spontaneous fluctuation area in order to reflect the spontaneous regional activity in the resting state. It has been widely viewed as a means to reveal the intrinsic brain activity alterations in addiction. In the current study, We found increased ALFF in the left PFC of smokers in comparison of non-smokers, which was in accordance with the findings of previous fMRI studies (Wen et al., 2021). The PFC plays an important role in reward evaluation and is the basis of working memory, which stores smoking-related information and enables rapid integration and updating of other information to guide target behaviors (Hyman et al., 2006). Keeping goal representations within the PFC is vital to the cognitive control that enables goal-directed behavior to succeed (Miller, 2000; Rolls, 2004). It has been reported that the prefrontal lobe is selectively damaged by cigarette smoking (Franklin et al., 2007). The PFC not only plays a positive role in guiding organisms to achieve their goals successfully, but also plays an important role in inhibiting maladaptive responses (Hyman et al., 2006). As a result of the effects of nicotine on the PFC, smokers may have difficulty resisting the urge to smoke even though they know the dangers of smoking due to pathological valuations and a lack of top-down behavioral control.

In addition, we also noted that compared with non-smokers, smokers showed decreased spontaneous brain activity in right calcarine sulcus as well as attenuated functional connectivity between the left mSFG with visual attention areas including the left calcarine sulcus, lingual gyrus and fusiform gyrus. The mSFG is anatomically mostly associated with the anterior cingulate cortex (ACC) and the medial cingulate cortex (MCC) (Li et al., 2013) and that it is functionally related to cognitive control including conflict monitoring (Sohn et al., 2007; Ursu et al., 2009), error detection (Gehring and Fencsik, 2001; Pourtois et al., 2010), attention control (Crottaz-Herbette and Menon, 2006; Luo et al., 2007). Visual information integration and attention processing are important functions of the calcarine sulcus, which is part of the visual attention network (Bezdek et al., 2015). Yang et al. (2022) has found decreased static functional connectivity density in the calcarine sulcus in nicotine dependent individuals. Fusiform gyrus served as a vital component of the visual cortex and was involved in a range of visual cognitive functions, including face and body recognition, color information processing and emotion perception in facial stimulation (Sergent et al., 1992; Kanwisher et al., 1997; Wang et al., 2013; Weiner et al., 2014). One study found greater neural activation in fusiform gyrus in smokers after exposure to smoking-related images than neutral images (Due et al., 2002). And it has been demonstrated by a meta-analysis that subjects who are addicted to tobacco, alcohol, and cocaine have a higher gray matter volume in the fusiform gyrus (Zhang et al., 2021). The reduced functional connectivity between the mSFG and the visual attention areas in our results may suggest that neural communication between the cognitive control network and the visual attention network is impaired in chronic smokers. Top-down attentional bias mechanism in visual cortex has been widely reported (Buschschulte et al., 2014). Previous studies have found that smokers show top-down attentional bias disorder and inhibitory attentional bias disorder to cigarette cues (Marks et al., 2016; Wilcockson et al., 2021). Our results suggested that the reduced functional connectivity of these regions is connected with the attentional bias of smokers, which may make it easier for them to collect smoking-related information, thus inducing craving and even smoking behaviors.

Located between the calcarine sulcus and the fusiform gyrus, the lingual gyrus plays a crucial role in visual processing, and the collateral sulcus reaches the temporal lobe, which links the PHG (Jung et al., 2014). There have been research suggesting that the PHG plays an important role in episodic memory, associative memory, and recollection (Zola-Morgan et al., 1989; Davachi et al., 2003; Diana et al., 2010), as well as visuospatial processing (Epstein and Kanwisher, 1998; Ekstrom et al., 2003; Stevens et al., 2012). Smoking cues (such as seeing someone smoking or cigarettes related cues) can trigger recall of smoking-related memories in smokers. An fMRI smoking cue-induced task study found that activation of the PHG was inversely correlated with the severity of nicotine dependence (McClernon et al., 2008). In the current study, functional connectivity between mSFG and lingual gyrus as well as PHG was decreased, and it was negatively correlated with FTND scores. These reflected a disruption of the integrity of functional connectivity between cognitive control and visual episodic memory in smokers, and that the greater the nicotine dependence, the weaker the functional connectivity.

We also noticed that cigarette smoking had a particularly prominent effect on the functional connectivity of precuneus. The precuneus, involved in personal value attribution and emotion regulation (Zhang and Volkow, 2019), is thought to be an important component of the default mode network (DMN). Increasing evidence suggested that deregistration of DMN and interactions with other networks in substance use disorder (SUD) impairs the cognitive and affective processes that lead to craving and relapse (Li et al., 2014; Ding et al., 2015; Wang et al., 2016; Hackshaw et al., 2018). Previous studies have shown that reduced spontaneous neural activity in the precuneus is associated with long-term smoking (Wang et al., 2017). As subjects examined episodic memory, understood thoughts or behaviors of other people, appraised emotional information, and reflected on themselves, the DMN was actively engaged (Buckner et al., 2008; Spreng et al., 2009; Andrews-Hanna, 2012). The reduced functional connectivity between SFG and the precuneus in the current study may indicate that the subconscious of smokers is unable to identify the significance of external or internal factors that are influential to themselves, such as feedback on continuous smoking behavior (Konova et al., 2013). Emotional regulated cognitive control and decision-making in smokers may be affected by lower connectivity in these areas, meaning that smoking is uncontrolled despite knowing the negative consequences.

Another interesting result of this study was the attenuated functional connectivity between SFG, mSFG and cerebellar subregions including cerebellum 4 5, cerebellum 6 and cerebellum 8. The neural basis of cigarette smoking was thought to be mainly related to dopaminergic neural circuits composed of the prefrontal cortex, insula, striatum and so on (Yang et al., 2020). While the cerebellum is widely believed to be vital for coordinating movement, maintaining posture and balance, there is growing evidence that its role in emotional regulation, attentional processing and decision making cannot be ignored (Sathyanesan et al., 2019). The cerebellum has a variety of nicotinic acetylcholine receptor (nAChR) subtypes that are sensitive to nicotine sensitization (Paterson and Nordberg, 2000; Turner and Kellar, 2005). The excitotoxic effects of nicotine can cause cell loss in the cerebellum, especially Purkinje and granular neurons (Chen et al., 2003). Durazzo et al. (2004) found the effect of long-term smoking on choline concentrations in the cerebellum. The cerebellum has also been thought to be part of the addiction circuit (Miquel et al., 2016). Miquel et al found that drug-induced functional and structural alterations in the cerebellum were central to the transition from a pattern of recreational drug use to a compulsive behavioral phenotype (Miquel et al., 2009). In the cerebellum, several subregions are capable of projecting (receiving) signals to (from) the cerebral cortex (Bostan et al., 2013). According to cerebellar networks theory, interactions with the cerebral cortex may represent neural mechanism for cerebellar involvement in cue-induced craving and addiction (Bostan et al., 2013). Our results suggested that some cerebellar subregions (such as cerebellum 4 5, cerebellum 6 and cerebellum 8) are functionally connected to brain regions involved in cognitive and executive control (SFG and mSFG). As a consequence, attenuated functional connectivity between SFG, mSFG and cerebellar subregions might contribute to dysfunction associated with chronic smokers.

There are still several limitations in this study. First and foremost, the sample size of the study is relatively small, which may reduce the strength of our results. Secondly, previous studies have shown gender and racial differences in brain function in smokers (Bagga et al., 2018; McCarthy et al., 2019; Lin et al., 2021). The participants included in the current study were all Chinese males, so the results of this study may not account for all smokers. Finally, this study was cross-sectional. We will use longitudinal data to confirm and complement the brain functional alterations in smokers in the future.

## 5. Conclusion

In summary, our study combined amplitude of low frequency fluctuation (ALFF) and resting-state functional connectivity (rs-FC) to identify the alterations of intrinsic brain activity and neural connectivity abnormalities in smokers. The present study found increased ALFF in SFG with reduced functional connectivity to visual attention areas including the calcarine sulcus, lingual gyrus and fusiform gyrus, indicating impaired neural communication between the cognitive control function and the visual attention network, which perhaps played a significant role in the maintenance of cigarette smoking. Besides, our findings noted the role of the cerebellum in tobacco addiction extending previous findings that have primarily focused on changes in cerebrum. Overall, combing two different functional indicators to probe the alterations of neural activity in the resting state of smokers could provide a deeper insight into the neural mechanism of cigarette smoking.

## Data availability statement

The datasets presented in this article are not readily available because of the privacy of all the participants. Requests to access the datasets should be directed to YZ, zzuzhangyong2013@163.com.

## Ethics statement

The studies involving human participants were reviewed and approved by the Local Medical Ethics Committee of the First Affiliated Hospital of Zhengzhou University. The patients/participants provided their written informed consent to participate in this study.

## Author contributions

XN, YZ, XG, MZ, and QL conceived and designed the study. XN, XG, MZ, JD, and JS collected the data. XN analyzed the data, performed the statistical study, and drafted the manuscript. YZ, JC, SH, QL, and YW revised the manuscript. All authors contributed to the article and approved the submitted version.
